# Path loss modeling and performance evaluation of double IRSs-aided wireless communication systems based on spatial scattering channel model

**DOI:** 10.1038/s41598-023-34562-5

**Published:** 2023-05-24

**Authors:** Jihong Wang, Hao Ni

**Affiliations:** School of Electrical Engineering, Northeast Electric Power University, Jilin, 132012 China

**Keywords:** Information technology, Electrical and electronic engineering

## Abstract

Intelligent reflecting surface (IRS) is a key enabling technology to reshape the electromagnetic propagation environment and enhance the communication performance. Current single IRS-aided or multiple distributed IRSs-aided wireless communication systems leave inter-IRSs collaboration out of consideration, and as a result, the system performance may be severely restricted. For cooperative double IRSs-aided wireless communication systems, dyadic backscatter channel model is widely used in the performance analysis and optimization. However, the impact of factors such as the size and gain of IRS elements is omitted. As a result, the performance quantification and evaluation are inaccurate. In order to avoid the above limitations, spatial scattering channel model is leveraged to quantify the path loss of the double reflection link in typical application scenarios of double IRSs-aided wireless communication systems. When the near-field condition is satisfied, the electromagnetic wave signal transmitted between IRSs is a spherical wave, which leads to high-rank channel and a lower signal to noise ratio. This paper considers the rank-1 inter-IRSs equivalent channel and derives the closed-form received signal power which reveals its relationship with the deployment of IRSs and the physical and electromagnetic properties of IRSs. Taking the impact of near/far-field effects of IRS on signal propagation further into consideration, the network configurations under which double cooperative IRSs can enhance the system performance are recognized. Simulation results show that whether double IRSs should be selected to assist in the communication between the transmitter and the receiver depends on practical network configurations, and the same number of elements should be assigned to the two IRSs to maximize the system performance if they are adopted.

## Introduction

As a key enabling technology for the sixth generation (6G) wireless communication systems, intelligent reflecting surface (IRS) is capable of reshaping the electromagnetic propagation environment and dramatically enhancing the communication performance by smartly tuning the amplitude and/or the phase shift of the incident electromagnetic wave via a large number of low-cost elements integrated on it^1,2^. Different from conventional active relays which require bulky transmit radio frequency chains, IRS acts as a passive array and directly reflects the incident signal, and its low power property conforms to the development tendency of future communication technologies^3^.

However, most of the existing research focuses on single IRS-aided wireless communication systems and multiple distributed IRSs-aided wireless communication systems without considering the impact of inter-IRSs collaboration, which may lead to inferior system performance. Although IRS can enhance the end-to-end communication by creating virtual connections, the equivalent path loss of the cascaded base station (BS)-IRS-user link is the product (instead of the sum) of the path losses of the BS-IRS and IRS-user sub-links in single IRS-aided wireless communication systems. In order to improve the IRS array gain to a reasonable level, a large number of elements are required to compensate for the path loss caused by the multiplicative fading effects^4^. Therefore, the academia and industry explore to utilize double IRSs to assist the communication between the BS and the user to further enhance the system performance^5,6^. In this case, the equivalent path loss of the double reflection link (BS-IRS1-IRS2-user) is the product of the path losses of the BS-IRS, inter-IRSs and IRS-user sub-links, and the system performance is also constrained by multiplicative fading effects. Reasonable deployment of double IRSs and taking advantage of the rich scattering environment created by IRSs are helpful in reducing the path loss of double IRSs-aided wireless communication systems. However, whether cooperative double IRSs-aided wireless communication system is superior to its single counterpart requires in-depth investigation.

**Figure 1:**
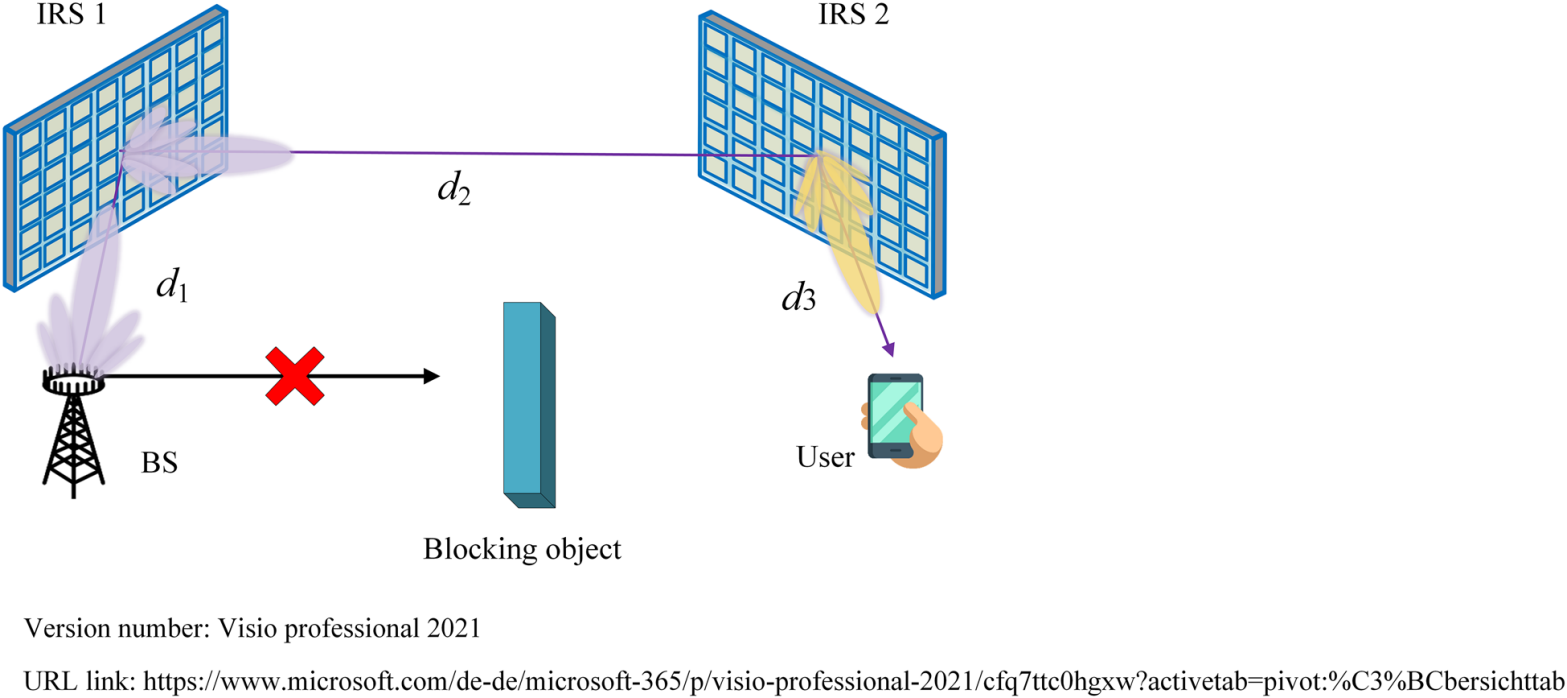
Typical application scenario of double IRSs-aided wireless communication systems (only the double reflection link is included).

Accurate channel characterization and modeling is an important basis for the performance analysis and optimization of IRS-aided wireless communication systems. In general, there are mainly two techniques to model the cascaded channel via IRS, i.e., dyadic backscatter channel model and spatial scattering channel model^7^. Dyadic backscatter channel model is widely used in the performance analysis and optimization of IRS-aided wireless communication systems, and the impact of IRS on signal propagation is modeled as a diagonal matrix. The linear independence between signals reflected by neighboring IRS elements will lead to inaccurate system performance evaluation^8^. By taking the direction of arrival (DoA) and angle of arrival (AoA) of the incident signal, the size and gain of IRS elements into account, spatial scattering channel model can help quantify the cascaded path loss of IRS-aided wireless communication systems accurately^9^. However, current spatial scattering channel model-based path loss modeling developed for single IRS-aided wireless communication systems^9^ cannot effectively quantify the path loss of the double reflection link. To the best of the authors’ knowledge, no work has been done to quantify the path loss of double IRSs-aided wireless communication systems by applying spatial scattering channel model, even for simple free-space propagation. Therefore, specialized spatial scattering channel model-based path loss modeling for double IRSs-aided wireless communication systems is urgently needed to lay necessary foundations for the performance analysis and optimization of such systems. This motivates us to develop a closed-form path loss of the double reflection link based on spatial scattering channel modeling for the typical application scenario of double IRSs-aided wireless communication systems shown in Fig. 1, that is, the BS can only communicate with the user through the double reflection link, and other links are severely blocked by obstacles. The research results provide theoretical basis for the performance analysis of double IRSs-aided wireless communication systems. The innovations of this paper are summarized as follows:

- To avoid the limitations of dyadic backscatter channel model, spatial scattering channel model is firstly leveraged to quantify the cascaded path loss in cooperative double IRSs-aided wireless communication system. The relationship between the received power at the user and various system parameters such as the transmit power of the BS, the gains of the transmit antenna and the receiving antenna, the number of IRS elements, the size and gain of each IRS element, the carrier wavelength and the deployment of IRSs is revealed.
- Taking the near/far-field effects of IRS further into account, the above cascaded path loss model is adopted to evaluate the performance of cooperative double IRSs-aided wireless communication system. The network configurations under which double cooperative IRSs can effectively enhance the system performance are recognized. In addition, the same number of elements should be assigned to the two IRSs to maximize the received signal to noise ratio (SNR) at the user, the channel capacity and minimize bit error rate (BER) if they are adopted. The optimal positions of double IRSs in far-field case are also identified to achieve the maximum received SNR at the user.

## Related works

Based on different assumptions and application scenarios, a substantial number of studies leverage different channel models to evaluate the performance gains achieved by single IRS- or double IRSs-aided wireless communication systems, and the relevant works are briefly summarized in Table 1. Dyadic backscatter channel model is widely used in the performance analysis and optimization of IRS-aided wireless communication systems, and its general model is summarized as below:

1 $${\mathbf{H}}={\mathbf{H}}_{r} {\boldsymbol{\Theta}\mathbf{ H}}_{t}$$

where **H**_*r*_ and **H**_*t*_ denote the equivalent channel matrices from the IRS to the receiver and from the transmitter to the IRS, respectively. The impact of IRS on signal propagation is modeled as a diagonal matrix. The linear independence between signals reflected by neighboring IRS elements will lead to inaccurate system performance evaluation of IRS-aided wireless communication systems^8^. In addition, the existing works usually assume that each item in the channel matrix obeys certain statistical distribution, such as the Rician distribution^10^. In general, the difference from the actual channel distribution is known to have a negative impact on system performance^7^.

**Table 1:** Characteristics analysis and performance comparison of state-of-the-art channel models for IRS-aided wireless communication systems.

Refs.	Network configurations	Channel model	Features
^11–14^	Single IRS	Dyadic backscatter	(1) Widely used in system analysis and optimization (2) Ignore the IRS properties (3) Rarely consider the near-field and far-field cases (4) AoA and DoA are omitted
^15^	Single IRS	Spatial scattering	(1) Ignore the IRS properties (2) AoA and DoA are left out of consideration
^16^	Single IRS	Spatial scattering	Ignore the IRS properties
^17^	Single IRS	Spatial scattering	Lack accurate description of the characteristics of IRS elements
^18^	Passive reflector	Spatial scattering	Passive reflectors are inapplicable to IRS
^9,19^	Single IRS	Spatial scattering	(1) Consider the IRS properties (2) Suitable for both near-field and far-field cases (3) AoA and DoA are involved
^20^	Single IRS	Spatial scattering	The mutual impedance theory is inapplicable to planar IRS
^21–23^	Double IRSs	Dyadic backscatter	(1) Widely used in system analysis and optimization (2) Ignore the IRS properties (3) Suitable for the far-field case
^24^	Double IRSs	Saleh-Valenzuela	Ignore the IRS properties
^25^	Multiple distributed IRSs	Dyadic backscatter	(1) Widely used in system analysis and optimization (2) Ignore the IRS properties (3) Rarely consider the near-field and far-field cases (4) AoA and DoA are ignored (5) Inter-IRSs cooperation is omitted
^26^	Multiple IRSs	Dyadic backscatter	(1) Inter-IRS cooperation is considered (2) Ignore the IRS properties (3) Rarely consider the near-field and far-field cases (4) AoA and DoA are not taken into account (5) Widely used in system analysis and optimization
Ours	Double IRSs	Spatial scattering	(1) IRS properties are taken into consideration (2) Suitable for both the near-field and far-field cases (3) AoA and DoA are involved

On the basis of dyadic backscatter channel model, alternating optimization (AO) and successive convex approximation (SCA) algorithms are used by^11^ to solve the confidentiality maximization problem with hardware constraints in IRS-aided millimeter wave (mmWave) communication system. The received signal at the user is shown in Eq. (2):

2 $$y={\varvec{h}}^{{\text{H}}} {{\varvec{\Theta}}}{\varvec{GF}}_{RF} \mathcal{Q}\left( {{\varvec{\omega}}s} \right) + n$$

where ***h***^H^ denotes the IRS-user channel matrix; ${\boldsymbol{\Theta}}$ denotes the IRS reflection coefficient matrix; ***G*** denotes the AP-IRS channel matrix; ***F***_*RF*_ denotes the analog beamforming codebook; ${\boldsymbol{\omega}}$ denotes the digital beamforming vector; *s* denotes the transmit signal; $\mathcal{Q}$(⋅) denotes the 1-bit quantizer, and *n* denotes the additive Gaussian white noise. Fractional programming and AO algorithms are used by^12^ to solve the problem of maximizing the energy efficiency of IRS-aided multicast communication system, and the *k*^th^ (*k*  = 1,…, *K*) received signal of the mobile user is:

3 $$y_{k} = {\mathbf{t}}_{k}^{{\text{H}}} {{\varvec{\Phi}}}{\mathbf{Hs}} + {\mathbf{g}}_{k}^{{\text{H}}} {\mathbf{s}} + z_{k}$$

where **H** denotes the channel matrix from the BS to the IRS; ${\boldsymbol{\Phi}}$ is a diagonal matrix which denotes the effective phase shifts adopted by all the reflecting elements on the IRS; ${\mathbf{t}}_{k}^{{\text{H}}}$ denotes the channel vector from the IRS to the *k*^th^ mobile user; ${\mathbf{g}}_{k}^{{\text{H}}}$ denotes the BS to the user channel vector, and *z*_*k*_ denotes the cyclic symmetric complex Gaussian noise with zero mean and unit variance at the *k*^th^ mobile user. In^13^, maximizing the achievable rate of the IRS-aided mmWave non-orthogonal multiple access (NOMA) system while satisfying the user’s minimum rate and transmit power constraints is divided into three sub-optimization problems, i.e., power allocation, joint phase shifts and analog beamforming optimization, and digital beamforming design, then solved by using alternating manifold optimization and SCA. The received signal at the *k*^th^ user in the *n*^*th*^ (*n* = 1,…, *N*) group is:

4 $$y_{n,k} = {\varvec{h}}_{n,k}^{{\text{H}}} \Theta {\varvec{GFWPs}} + \mu_{n,k}$$

where ***P*** denotes the power allocation matrix; ***W*** denotes the digital beamforming matrix; ***F*** denotes the analog beamforming matrix; ***h***_*n*,*k*_ denotes the channel vector from IRS to the *k*^th^ user in the *n*^th^ group; *μ*_*n*,*k*_ denotes the noise at the *k*^th^ user in the *n*^th^ group. Block coordinate descent algorithm is used by^14^ to optimize the AP transmit beamforming vector and the IRS passive beamforming in IRS-aided broadcast network with power splitting to ensure the users’ quality of service and self-sustainability of the IRS. The received signal at the *k*^th^ user can be expressed as:

5 $$y_{k} = {\mathbf{h}}_{d,k}^{{\text{H}}} {{\varvec{\upomega}}}x + {\mathbf{h}}_{r,k}^{{\text{H}}} \boldsymbol{\Psi} \mathbf{G}{{\varvec{\upomega}}}x + n_{k}$$

where ${\mathbf{h}}_{d,k}^{{\text{H}}}$ denotes the channel vector from AP to the *k*^th^ user; ${\mathbf{h}}_{r,k}^{{\text{H}}}$ denotes the channel vector from IRS to the *k*^th^ user; ${{\varvec{\upomega}}}$ denotes the transmit beamforming vector; *x* denotes the transmitted signal, and *n*_*k*_ denotes the Gaussian white noise at the *k*^th^ user.

Spatial scattering channel model can avoid the limitations of dyadic backscatter channel model and better demonstrate the propagation mechanism through an IRS. To be specific, each IRS element is regarded as a reflector in the environment creating a distinct propagation path. Therefore, the cascaded channel via IRS (with *Q* elements) is the superposition of all paths, as shown in Eq. (6).

6 $${\mathbf{H}} = \sum\limits_{q = 1}^{Q} {\alpha_{q} \Gamma_{q} } {{\varvec{\upalpha}}}_{{\mathbf{R}}} \left( {\theta_{R,q} ,\varphi_{R,q} } \right){{\varvec{\upalpha}}}_{{\mathbf{T}}}^{{\text{H}}} \left( {\theta_{T,q} ,\varphi_{T,q} } \right)$$

where *α*_*q*_ is the channel gain excluding the effects of element *q*. Γ_*q*_ is the control effect of element *q*. ${{\varvec{\upalpha}}}_{{\mathbf{R}}}$ and ${{\varvec{\upalpha}}}_{{\mathbf{T}}}$ are the array steering vectors at the receiver and the transmitter, respectively. *θ*_*R*,*q*_ and *φ*_*R*,*q*_ are the elevation and azimuth angles of element *q* with respect to the receiver. Similarly, *θ*_*T*,*q*_ and *φ*_*T*,*q*_ are defined for element *q* with respect to the transmitter. Assuming that an IRS with *Q* reflecting elements is deployed on the ground plane^15^, and the IRS is regarded as a specular reflector. Therefore, the total received power from the direct link and cascaded reflection link via IRS is:

7 $$P_{r} \approx (Q + 1)^{2} \times P_{t} \times \left(\frac{\lambda }{{4{\uppi }(d_{1} + d_{2} )}}\right)^{2}$$

where *P*_*t*_ is the transmission power;* λ* denotes the carrier wavelength; *d*_1_ and *d*_2_ are the distance from the IRS to the transmitter and the receiver, respectively. From Eq. (7), it can be seen that the received power is inversely proportional to the square of the length of the cascaded reflection link via IRS, i.e., (*d*_1_ + *d*_2_)^2^. However, this conjecture is disproven by^16^ and it might hold for an infinitely large IRS or the near-field case. Based on physical optics techniques, the following path loss model is proposed in^16^:

8 $$\begin{aligned} PL & = \frac{{(4\pi d_{1} d_{2} )^{2} }}{{G_{t} G_{r} (XY)^{2} \cos (\theta_{i} )^{2} \text{sinc}\left( {\frac{\pi Y}{\lambda }(\sin \theta_{s} - \sin \theta_{r} )} \right)}} \\ & \mathop = \limits^{(a)} \frac{{(4\pi d_{1} d_{2} )^{2} }}{{G_{t} G_{r} (XY)^{2} (\cos \theta_{i} )^{2} }} \\ \end{aligned}$$

where *G*_*t*_ and *G*_*r*_ are the gains of the transmit antenna and the receiving antenna, respectively;* X* × *Y* is the physical size of IRS; *θ*_*i*_, *θ*_*s*_ and *θ*_*r*_ are the incident angle from the transmitter to the IRS, the observation angle and the desired reflection angle, respectively. (a) follows when *θ*_*s*_  = *θ*_*r*_. Equation (8) indicates that the path loss is positively proportional to (*d*_1_*d*_2_)^2^ instead of (*d*_1_ + *d*_2_)^2^. It also explains why the surface consists of many elements that individually act as diffuse scatters can jointly beamform the signal in a desired direction with a certain beamwidth. The radiation density based on the scattered electric field intensity in the near field of IRS is calculated by^17^. The path loss is described as a function of the Euclidean distance from the transmit antenna to each element on the IRS, the wave numbers, the element impedance, the input antenna current, and the radiation vector generated by the current. However, the properties of the IRS elements are not involved. Based on the far-field received power model of a metal reflector, the optimal received power of a passive reflector-enhanced non-line-of-sight (NLOS) link in the mmWave band is derived by^18^, as shown in Eq. (9).

9 $$P_{r} = \frac{{P_{t} G_{t} a^{2} b^{2} }}{{\left( {4{\uppi }} \right)^{2} r_{1}^{2} r_{2}^{2} }}\cos^{2} \left( {\theta_{i} } \right)\left( {\frac{{\sin \left( {\frac{{{\uppi }b}}{\lambda }\left( {\sin \left( {\theta_{r} } \right) - \sin \left( {\theta_{i} } \right)} \right)} \right)}}{{\frac{{{\uppi }b}}{\lambda }\left( {\sin \left( {\theta_{r} } \right) - \sin \left( {\theta_{i} } \right)} \right)}}} \right)^{2}$$

where *a* × *b* denotes the size of passive reflector; *r*_1_ and *r*_2_ denote the Euclidean distances from the transmit antenna to the passive reflector and from the passive reflector to the receiving antenna, respectively. However, since passive reflector is different from the IRS, the receive power model is not applicable to IRS-aided wireless communication systems. By studying the physical and electromagnetic properties of IRS, a general path loss model is developed for single IRS-aided wireless communication systems, and the received signal power is shown in Eq. (10)^9^.

10 $$P_{r} = \frac{{P_{t} G_{t} G_{r} Gd_{x} d_{y} \lambda^{2} }}{{64{\uppi }^{3} }}\left| {\sum\limits_{{m = 1 - \frac{M}{2}}}^{\frac{M}{2}} {\sum\limits_{{n = 1 - \frac{N}{2}}}^{\frac{N}{2}} {\frac{{\sqrt {F_{n,m}^{combine} } \Gamma_{n,m} }}{{r_{n,m}^{t} r_{n,m}^{r} }}e^{{\frac{{ - j2{\uppi }\left( {r_{n,m}^{t} + r_{n,m}^{r} } \right)}}{\lambda }}} } } } \right|^{2}$$

where *G* and *d*_*x*_ × *d*_*y*_ are the gain and physical size of each IRS element, respectively. *N* and *M* are the number of rows and columns of elements which are regularly arranged on IRS. $F_{n,m}^{combine}$ accounts for the impact of the normalized power radiation patterns on the received signal power. Γ_*n*,*m*_ is the reflection coefficient of the IRS element in row *n* and column *m*, i.e., *U*_*n*,*m*_. $r_{n,m}^{t}$ and $r_{n,m}^{r}$ are the Euclidean distances from the transmitter and receiver to *U*_*n*,*m*_, respectively. To extend the application scenarios of IRS, angle-dependent loss factor is formulated to quantify the impact of antenna’s direction of the transmitter, receiver and IRS elements on the path loss, and the path loss model proposed in^9^ is refined for IRS-aided wireless communication systems operating in the mmWave band^19^. In addition, the relationship between the scattering gain of an IRS element and its physical size is derived, as shown in Eq. (11).

11 $$G = \frac{{4{\uppi }d_{x} d_{y} }}{{\lambda^{2} }}$$

The path loss model proposed by^20^ based on the IRS elements mutual impedance theory in far-field case is not applicable to planar IRS^19^.

Current research results show that rationally designed double IRSs-aided wireless communication systems outperform single IRS-aided wireless communication systems. To be specific, double IRSs-aided wireless communication systems are considered for the first time in^21^. Under the assumptions that other links are severely blocked and the inter-IRSs channel is of rank 1, the passive beamforming design problem is solved. The geometric relationship between the two IRSs is exploited to obtain the power gain of the user, as shown in Eq. (12).

12 $$\left| H \right|^{{2}} \approx \frac{{\alpha^{{3}} }}{{\left( {d_{r} d_{s} d_{t} } \right)^{2} }}\left( {K_{1} K_{2} } \right)^{2}$$

where *H* is the channel gain of the cascaded reflection link. $\alpha/d_{r}^{2}$, $\alpha/d_{s}^{2}$ and $\alpha/d_{t}^{2}$ represent the approximate path losses between the BS and the elements on IRS 1, between the elements on IRS 1 and IRS 2 and between the elements on IRS 2 and the user, respectively. *K*_1_ and *K*_2_ are the number of elements on IRS 1 and IRS 2, respectively. Given the total number of IRS elements *K*, reasonable element assignment and reflection coefficient matrix design can achieve a power gain of order $\mathcal{O}$(*K*^4^). However, sufficient IRS elements are required to compensate for the multiplicative fading effects of the cascaded reflection link and guarantee their superior performance. The active beamforming at the BS and passive reflection beamforming at the two IRSs are jointly optimized for double IRSs-assisted multi-user multi-input multi-output (MIMO) system to maximize the minimum uplink signal-to-interference-plus-noise ratio of all users^22^. The channel model shown in Eq. (13) is applied, i.e., apart from the double reflection link, two single reflection links BS-IRS 1-user and BS-IRS 2-user are further taken into consideration to enhance the spatial multiplexing gain of double IRSs-assisted wireless communication systems.

13 $$H_{q} = G_{2} \Phi_{2} D\Phi_{1} u_{1,q} + G_{2} \Phi_{2} u_{2,q} + G_{1} \Phi_{1} u_{1,q}$$

where *H*_*q*_ is the superimposed uplink channel for user *q*. *u*_1,*q*_ and *u*_2,*q*_ are the baseband equivalent channels for the user *q*-IRS 1 and user *q*-IRS 2 links, respectively. Φ_1_ and Φ_2_ are the diagonal reflection matrices which model the impact of IRS 1 and IRS 2 on signal propagation, respectively. *G*_1_ and *G*_2_ are the baseband equivalent channels for the IRS 1-BS and IRS 2-BS links, respectively. *D* is the baseband equivalent channel for the IRS 1-IRS 2 link. Based on the same channel model, the impact of array response between the transmit antenna/receiving antenna and IRS is further considered in^23^. The transmit covariance matrix and the passive beamforming matrices of the two cooperative IRSs are jointly optimized to maximize the channel capacity of double IRSs-aided single user MIMO system. By further analyzing the correlation between the array responses of the BS-IRS 1, BS-IRS 2, IRS 1-user and IRS 2-user channels, the closed-form channel capacity is derived for double IRSs-aided single user MIMO system with rank-1 and rank-2 channels. Simulation results show that double IRSs-aided MIMO system can achieve a channel capacity of order $\mathcal{O}$(*M*^4^) with an asymptotically large *M* (the total number of IRS elements). The extended Saleh-Valenzuela channel model in Eq. (14) is adopted by^24^, and with the objective of maximizing the weighted sum rate of downlink transmissions, the digital precoding matrix at the BS and the analog phase shifters at the two IRSs are alternately optimized for double IRSs-aided multi-user MIMO system operating in the mmWave band.

14 $$H_{1} = \sum\limits_{q = 1}^{{N_{path} }} {\alpha_{q} {\mathbf{a}}_{{\mathbf{r}}} \left( {\psi_{q}^{r} ,\beta_{q}^{r} } \right){\mathbf{a}}_{{\mathbf{t}}} } \left( {\psi_{q}^{t} ,\beta_{q}^{t} } \right)^{{\text{H}}}$$

where *H*_1_ is the equivalent channel from the BS to IRS 1. *N*_*path*_ denotes the number of physical propagation paths between the BS and IRS 1. *α*_*q*_ is the channel gain of path *q*. **a**_**t**_ $\left( {\psi_{q}^{t} ,\beta_{q}^{t} } \right)$ and **a**_**r**_ $\left( {\psi_{q}^{r} ,\beta_{q}^{r} } \right)$ are the array response vectors of the transmit antenna and IRS associated with path *q*. $\psi_{q}^{t}$ and $\beta_{q}^{t}$ are the azimuth and elevation angles of departure of path *q*, respectively. $\psi_{q}^{r}$ and $\beta_{q}^{r}$ are the azimuth and elevation AoAs of path* q*, respectively. Based on the same channel model, the transmit beamforming matrix of the BS and the reflection coefficient matrices of the two IRSs are alternately optimized to maximize the weighted sum rate of a multi-IRS-aided multi-user MIMO system^25^. However, the above systems leave inter-IRSs collaboration out of consideration, and each IRS only serves the users in its half reflection space. Offline beam training solution is proposed in^26^, and the channel model shown in Eq. (15) is utilized to maximize the end-to-end channel gain of multi-IRS-aided wireless networks.

15 $$h_{0,J + 1} \left( {{\varvec{\Omega}}} \right) = {\varvec{g}}_{{a_{Q} ,J + 1}}^{{\text{H}}} {{\varvec{\Phi}}}_{{a_{Q} }} \left( {\prod\limits_{q = 1}^{q = Q - 1} {{\varvec{S}}_{{a_{q} ,a_{q + 1} }} {{\varvec{\Phi}}}_{{a_{q} }} } } \right){\varvec{H}}_{{0,a_{1} }} {\varvec{w}}_{B}$$

where *h*_0,*J*+1_ is the equivalent multi-hop BS-user channel. **Ω** represents the multi-hop reflection path between the BS and the user, and *Q* is the total number of IRSs on the path. ***w***_*B*_ is the precoding vector of the BS. $${\varvec{H}}_{{0,a_{1} }}$$ is the equivalent channel between the BS and its next-hop IRS. $${{\varvec{\Phi}}}_{{a_{q} }}$$ is the reflection coefficient matrix of IRS *q*. $${\varvec{S}}_{{a_{q} ,a_{q + 1} }}$$ represents the equivalent channel matrix between IRS *q* and its next-hop IRS. $${{\varvec{\Phi}}}_{{a_{Q} 
}}$$ denotes the reflection coefficient matrix of the last IRS, and $${\varvec{g}}_{{a_{Q} ,J + 1}}^{{\text{H}}}$$ is the equivalent channel from the last IRS to the user.

The above spatial scattering channel modeling-based path loss models effectively avoid the limitations of dyadic backscatter channel modeling. However, the results derived for single IRS-aided wireless communication systems cannot be directly extended to double IRSs-aided systems. Although there are research results based on Saleh-Valenzuela channel model which takes the impact of AoA and DoA into account, the physical and electromagnetic properties of IRS are still omitted. To the best of the authors’ knowledge, there is no relevant research on spatial scattering channel model-based path loss modeling for cooperative double IRSs-aided wireless communication networks, and this motivates our work in this paper. The research results in this paper lay indispensable foundations for future research on double IRSs-aided wireless communication systems.

### Spatial scattering channel model-based path loss modeling for double IRSs-aided wireless communication systems

**Figure 2:**
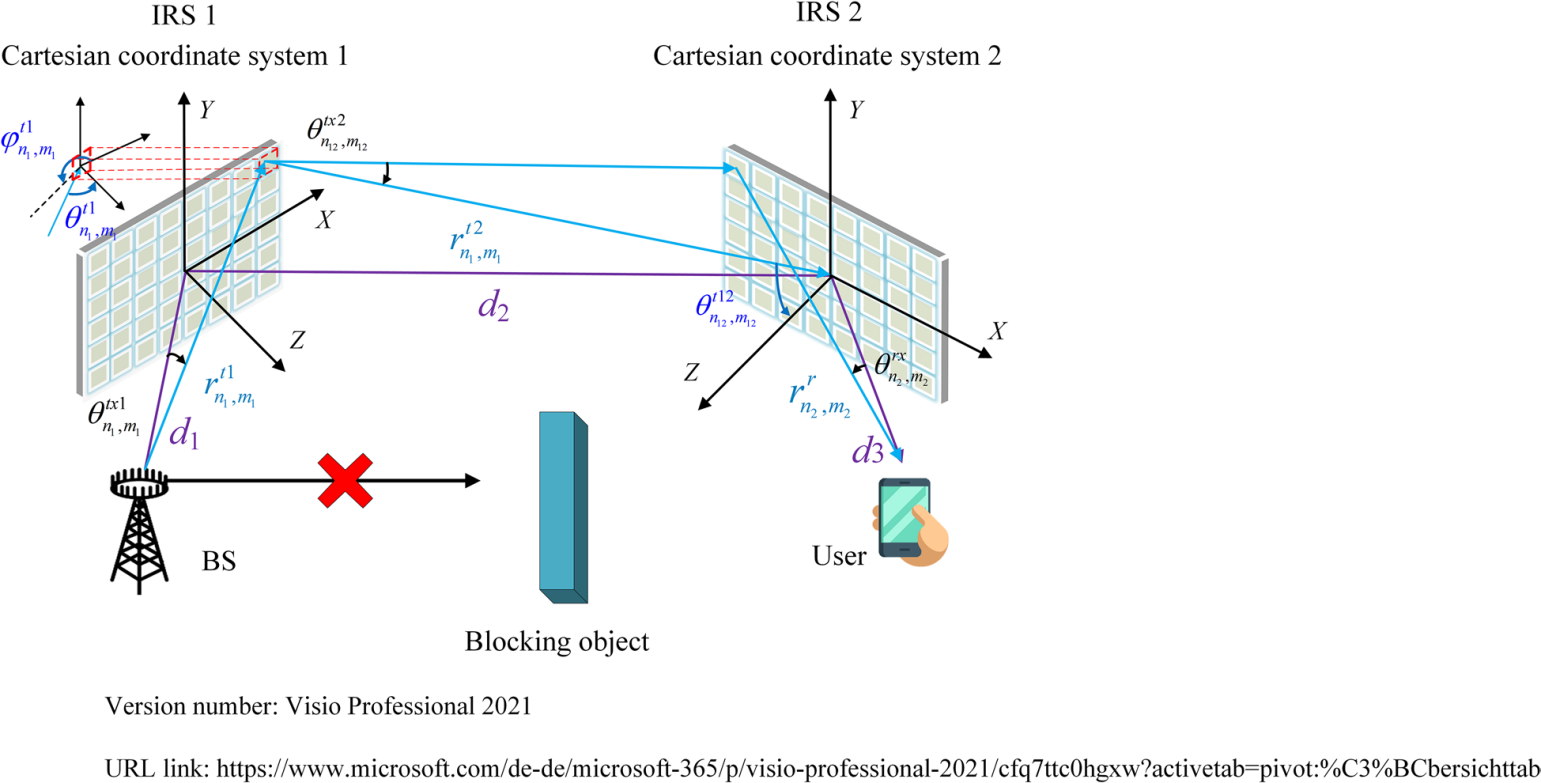
Spatial scattering channel model-based path loss modeling for the considered double IRSs-aided wireless communication systems.

As illustrated in Fig. 2, in order to minimize the path loss of the double reflection link, IRS 1 and IRS 2 are placed close to the BS and the user, respectively. They are placed in *X*–*Y* plane of Cartesian coordinate systems 1 and 2 whose origins align with the geometric centers of the two IRSs, respectively. *N*_1_ and *M*_1_ are the number of rows and columns of elements which are regularly arranged on IRS 1, and similarly, *N*_2_ and *M*_2_ are the number of rows and columns of elements on IRS 2. Without loss of generality, the above parameters are assumed to be even numbers. *d*_*x*_ × *d*_*y*_ is the size of each IRS element. *U*(*n*_1_,*m*_1_) represents the element in row *n*_1_(*n*_1_ ∈ [− *N*_1_/2 + 1,*N*_1_/2]) and column *m*_1_(*m*_1_ ∈ [− *M*_1_/2 + 1,*M*_1_/2]) on IRS 1, and its center coordinate in Cartesian coordinate system 1 is ((*m*_1_ − 1/2)*d*_*x*_,(*n*_1_ − 1/2)*d*_*y*_,0). Its programmable reflection coefficient is $\Gamma_{{n_{1} ,m_{1} }}$, and the gain is *G*_1_. Similarly, parameters *U*(*n*_2_,*m*_2_), ((*m*_2_ − 1/2)*d*_*x*_,(*n*_2_ − 1/2)*d*_*y*_,0), $\Gamma_{{n_{2} ,m_{2} }}$ and *G*_2_ are defined for element *U*(*n*_2_,*m*_2_) on IRS 2. *F*(*θ*,*φ*) is the inherent normalized power radiation pattern of IRS elements. *F*^*tx*^(*θ*,*φ*) and *F*^*rx*^(*θ*,*φ*) are the normalized power radiation patterns of the transmit antenna and the receiving antenna, respectively. *d*_1_, *d*_2_ and *d*_3_ are the Euclidean distances from the BS to the center of IRS 1, between the centers of IRS 1 and IRS 2, from the center of IRS 2 to the user, respectively. $r_{{n_{1} ,m_{1} }}^{t1}$, $r_{{n_{1} ,m_{1} }}^{t2}$ and $r_{{n_{2} ,m_{2} }}^{r}$ are the Euclidean distances from the BS to *U*(*n*_1_,*m*_1_), between *U*(*n*_1_,*m*_1_) and the center of IRS 2, from *U*(*n*_2_,*m*_2_) to the user, respectively. $\theta_{{n_{1} ,m_{1} }}^{t1}$ and $\varphi_{{n_{1} ,m_{1} }}^{t1}$ are the elevation and azimuth angles from *U*(*n*_1_,*m*_1_) to the BS, respectively. Similarly, parameters $\theta_{{n_{2} ,m_{2} }}^{r}$ and $\varphi_{{n_{2} ,m_{2} }}^{r}$ are defined for *U*(*n*_2_,*m*_2_) with respect to the user. $\theta_{{n_{12} ,m_{12} }}^{t2}$ and $\varphi_{{n_{12} ,m_{12} }}^{t2}$ are the elevation and azimuth angles from *U*(*n*_2_,*m*_2_) to *U*(*n*_1_,*m*_1_), respectively. $\theta_{{n_{12} ,m_{12} }}^{t12}$ and $\varphi_{{n_{12} ,m_{12} }}^{t12}$ are parameters defined for the center of IRS 2 with respect to *U*(*n*_1_,*m*_1_). $\theta_{{n_{1} ,m_{1} }}^{tx1}$ and $\varphi_{{n_{1} ,m_{1} }}^{tx1}$ are the elevation and azimuth angles from the transmit antenna of the BS to *U*(*n*_1_,*m*_1_), respectively. $\theta_{{n_{12} ,m_{12} }}^{tx2}$ and $\varphi_{{n_{12} ,m_{12} }}^{tx2}$ are the elevation and azimuth angles from *U*(*n*_1_,*m*_1_) to *U*(*n*_2_,*m*_2_). $\theta_{{n_{12} ,m_{12} }}^{tx12}$ and $\varphi_{{n_{12} ,m_{12} }}^{tx12}$ are the elevation and azimuth angles from *U*(*n*_1_,*m*_1_) to the center of IRS 2. $\theta_{{n_{2} ,m_{2} }}^{rx}$ and $\varphi_{{n_{2} ,m_{2} }}^{rx}$ are the elevation and azimuth angles from the receiving antenna of the user to *U*(*n*_2_,*m*_2_).

The received signal power of *U*(*n*_1_,*m*_1_) from the BS is:

16 $$P_{{n_{1} ,m_{1} }}^{in} = \frac{{P_{t} G_{t} F^{tx1} \left( {\theta_{{n_{1} ,m_{1} }}^{tx1} ,\varphi_{{n_{1} ,m_{1} }}^{tx1} } \right)F\left( {\theta_{{n_{1} ,m_{1} }}^{t1} ,\varphi_{{n_{1} ,m_{1} }}^{t1} } \right)d_{x} d_{y} }}{{4\pi {r_{{n_{1} ,m_{1} }}^{t1}} ^{2} }}$$

where *P*_*t*_ is the transmit power of the BS, and *G*_*t*_ is the gain of the transmit antenna.

The reflected signal power of *U*(*n*_1_,*m*_1_) is:

17 $$P_{{n_{1} ,m_{1} }}^{reflect1} = P_{{n_{1} ,m_{1} }}^{in} \left| {\Gamma_{{n_{1} ,m_{1} }}^{2} } \right|$$

If IRS 1 is in the far field of IRS 2, the propagation distance of the signal reflected by *U*(*n*_1_,*m*_1_) towards IRS 2 can be approximated as the Euclidean distance between *U*(*n*_1_,*m*_1_) and the center of IRS 2. In this case, the power of the reflected signal received by *U*(*n*_2_,*m*_2_) from *U*(*n*_1_,*m*_1_) is:

18 $$\begin{aligned}P_{{n_{1} ,m_{1} }}^{r} &= \frac{{P_{{n_{1} ,m_{1} }}^{reflect1} G_{1} F^{tx2} \left( {\theta_{{n_{12} ,m_{12} }}^{tx2} ,\varphi_{{n_{12} ,m_{12} }}^{tx2} } \right)F\left( {\theta_{{n_{12} ,m_{12} }}^{t2} ,\varphi_{{n_{12} ,m_{12} }}^{t2} } \right)d_{x} d_{y} }}{{4\pi {r_{{n_{1} ,m_{1} }}^{t2}}^{2} }} \\ & =\frac{{P_{t} G_{t} G_{1} \left| {\Gamma_{{n_{1} ,m_{1} }}^{2} } \right|d_{x}^{2} d_{y}^{2} }} {{16\pi^{2} {r_{{n_{1} ,m_{1} }}^{t1}}^{2} {r_{{n_{1} ,m_{1} }}^{t2}}^{2} }} \times F^{tx1} \left( {\theta_{{n_{1} ,m_{1} }}^{tx1} ,\varphi_{{n_{1} ,m_{1} }}^{tx1} } \right)F\left( {\theta_{{n_{1} ,m_{1} }}^{t1} ,\varphi_{{n_{1} ,m_{1} }}^{t1} } \right)F^{tx2} \left( {\theta_{{n_{12} ,m_{12} }}^{tx2} ,\varphi_{{n_{12} ,m_{12} }}^{tx2} } \right)F\left( {\theta_{{n_{12} ,m_{12} }}^{t2} ,\varphi_{{n_{12} ,m_{12} }}^{t2} } \right) \\ \end{aligned}$$

where *F*^*tx*2^($\theta_{{n_{12} ,m_{12} }}^{tx2}$,$\varphi_{{n_{12} ,m_{12} }}^{tx2}$) and *F*($\theta_{{n_{12} ,m_{12} }}^{t2}$,$\varphi_{{n_{12} ,m_{12} }}^{t2}$) are the normalized power radiation function of *U*(*n*_1_,*m*_1_) with respect to *U*(*n*_2_,*m*_2_) and the reversed normalized power radiation function, respectively.

The electric field of the reflected signal received by *U*(*n*_2_,*m*_2_) from *U*(*n*_1_,*m*_1_) is:

19 $$E_{{n_{1} ,m_{1} }}^{r} = \sqrt {\frac{{2Z_{0} P_{{n_{1} ,m_{1} }}^{r} }}{{d_{x} d_{y} }}} e^{{ - j\left( {\frac{{2{\uppi }}}{\lambda }r_{{n_{1} ,m_{1} }}^{t1} - \phi_{{n_{1} ,m_{1} }} + \frac{{2{\uppi }}}{\lambda }r_{{n_{1} ,m_{1} }}^{t2} } \right)}}$$

where $\phi_{{n_{1} ,m_{1} }}$ is the phase shift introduced by *U*(*n*_1_,*m*_1_) to the incident signal, and *Z*_0_ is the characteristic impedance of the air.

The total electric field of the reflected signal received by *U*(*n*_2_,*m*_2_) from all elements on IRS 1 is:

20 $$E^{r - IRS1} = \sum\limits_{{m_{1} = 1 - \frac{{M_{1} }}{2}}}^{{\frac{{M_{1} }}{2}}} {\sum\limits_{{n_{1} = 1 - \frac{{N_{1} }}{2}}}^{{\frac{{N_{1} }}{2}}} {E_{{n_{1} ,m_{1} }}^{r} } }$$

The signal power received by *U*(*n*_2_,*m*_2_) from IRS 1 is:

21 $$P_{{n_{2} ,m_{2} }}^{in} = \frac{{\left| {E^{r - IRS1} } \right|^{2} }}{{2Z_{0} }}d_{x} d_{y} = \frac{{P_{t} G_{t} G_{1} d_{x}^{2} d_{y}^{2} }}{{16{\uppi }^{2} }}\left| {\sum\limits_{{m_{1} = 1 - \frac{{M_{1} }}{2}}}^{{\frac{{M_{1} }}{2}}} {\sum\limits_{{n_{1} = 1 - \frac{{N_{1} }}{2}}}^{{\frac{{N_{1} }}{2}}} {\frac{{\sqrt {F_{{n_{1} ,m_{1} }}^{combine} } }}{{r_{{n_{1} ,m_{1} }}^{t1} r_{{n_{{1}} ,m_{{1}} }}^{t2} }}e^{{ - j\left( {\frac{{2{\uppi }}}{\lambda }r_{{n_{1} ,m_{{1}} }}^{t1} - \phi_{{n_{1} ,m_{1} }} + \frac{{2{\uppi }}}{\lambda }r_{{n_{{1}} ,m_{{1}} }}^{t2} } \right)}} } } } \right|^{2}$$

where $F_{{n_{1} ,m_{1} }}^{combine}$  =  *F*^*tx*1^($\theta_{{n_{1} ,m_{1} }}^{tx1}$,$\varphi_{{n_{1} ,m_{1} }}^{tx1}$)*F*($\theta_{{n_{1} ,m_{1} }}^{t1}$,$\varphi_{{n_{1} ,m_{1} }}^{t1}$)*F*^*tx*2^($\theta_{{n_{12} ,m_{12} }}^{tx2}$,$\varphi_{{n_{12} ,m_{12} }}^{tx2}$)*F*($\theta_{{n_{12} ,m_{12} }}^{t2}$,$\varphi_{{n_{12} ,m_{12} }}^{t2}$). According to^9^, when IRS 1 is in the far field of IRS 2 and all elements on IRS 1 adopt intelligent reflection, that is, *F*^*tx*2^($\theta_{{n_{12} ,m_{12} }}^{tx2}$,$\varphi_{{n_{12} ,m_{12} }}^{tx2}$)≈*F*^*tx*2^($\theta_{{n_{12} ,m_{12} }}^{tx12}$,$\varphi_{{n_{12} ,m_{12} }}^{tx12}$), *F*($\theta_{{n_{12} ,m_{12} }}^{t2}$,$\varphi_{{n_{12} ,m_{12} }}^{t2}$)≈*F*($\theta_{{n_{12} ,m_{12} }}^{t12}$,$\varphi_{{n_{12} ,m_{12} }}^{t12}$), $\phi_{{n_{1} ,m_{1} }}$  = 2π($r_{{n_{1} ,m_{1} }}^{t1}$ + $r_{{n_{{1}} ,m_{{1}} }}^{t2}$)/*λ*, the received signal power of *U*(*n*_2_,*m*_2_) is maximized, and the value is:

22 $$\begin{gathered} P_{{n_{2} ,m_{2} }}^{in - \max } = \frac{{P_{t} G_{t} G_{1} d_{x}^{2} d_{y}^{2} }}{{16{\uppi }^{2} }} \times \left| {\sum\limits_{{m_{1} = 1 - \frac{{M_{1} }}{2}}}^{{\frac{{M_{1} }}{2}}} {\sum\limits_{{n_{1} = 1 - \frac{{N_{1} }}{2}}}^{{\frac{{N_{1} }}{2}}} {\frac{{\sqrt {F^{tx1} \left( {\theta_{{n_{1} ,m_{1} }}^{tx1} ,\varphi_{{n_{1} ,m_{1} }}^{tx1} } \right)F\left( {\theta_{{n_{1} ,m_{1} }}^{t1} ,\varphi_{{n_{1} ,m_{1} }}^{t1} } \right)F^{tx2} \left( {\theta_{{n_{12} ,m_{12} }}^{tx12} ,\varphi_{{n_{12} ,m_{12} }}^{tx12} } \right)F\left( {\theta_{{n_{12} ,m_{12} }}^{t12} ,\varphi_{{n_{12} ,m_{12} }}^{t12} } \right)} }}{{r_{{n_{1} ,m_{1} }}^{t1} r_{{n_{1} ,m_{1} }}^{t2} }}} } } \right|^{2} \hfill \\ \end{gathered}$$

The reflected signal power of *U*(*n*_2_,*m*_2_) is:

23 $$P_{{n_{2} ,m_{2} }}^{reflect2} = P_{{n_{2} ,m_{2} }}^{in - \max } \left| {\Gamma_{{n_{2} ,m_{2} }}^{2} } \right|$$

The power of the reflected signal received by the user from *U*(*n*_2_,*m*_2_) is:

24 $$\begin{aligned}P_{{n_{2} ,m_{2} }}^{r} &= \frac{{P_{t} G_{t}G_{1} G_{2} d_{x}^{2} d_{y}^{2} \left| {\Gamma_{{n_{2} ,m_{2} }}^{2}} \right|A_{r} }}{{64\pi^{3} {r_{{n_{2} ,m_{2} }}^{r}}^{2} }} \times F^{rx} \left( {\theta_{{n_{2} ,m_{2} }}^{rx} ,\varphi_{{n_{2} ,m_{2} }}^{rx} } \right)F\left( {\theta_{{n_{2} ,m_{2} }}^{r} ,\varphi_{{n_{2} ,m_{2} }}^{r} } \right) \\ & \times \left| {\sum\limits_{{m_{1} = 1 - \frac{{M_{1} }}{2}}}^{{\frac{{M_{1} }}{2}}} {\sum\limits_{{n_{1} = 1 - \frac{{N_{1} }}{2}}}^{{\frac{{N_{1} }}{2}}} {\frac{{\sqrt {F^{tx1} \left( {\theta_{{n_{1} ,m_{1} }}^{tx1} ,\varphi_{{n_{1} ,m_{1} }}^{tx1} } \right)F\left( {\theta_{{n_{1} ,m_{1} }}^{t1} ,\varphi_{{n_{1} ,m_{1} }}^{t1} } \right)F^{tx2} \left( {\theta_{{n_{12} ,m_{12} }}^{tx12} ,\varphi_{{n_{12} ,m_{12} }}^{tx12} } \right)F\left( {\theta_{{n_{12} ,m_{12} }}^{t12} ,\varphi_{{n_{12} ,m_{12} }}^{t12} } \right)} }}{{r_{{n_{1} ,m_{1} }}^{t1} r_{{n_{1} ,m_{1} }}^{t2} }}} } } \right|^{2} \\ \end{aligned}$$

where *A*_*r*_ is the effective area of the receiving antenna.

The electric field of the signal received by the user from *U*(*n*_2_,*m*_2_) is:

25 $$E_{{n_{2} ,m_{2} }}^{r} = \sqrt {\frac{{2Z_{0} P_{{n_{2} ,m_{2} }}^{r} }}{{A_{r} }}} e^{{ - j\left( {\frac{{2{\uppi }}}{\lambda }r_{{n_{2} ,m_{2} }}^{r} - \phi_{{n_{2} ,m_{2} }} } \right)}}$$

where $\phi_{{n_{2} ,m_{2} }}$ is the phase shift introduced by* U*(*n*_2_,*m*_2_) to the incident signal.

The total electric field of the reflected signal received by the user from all elements on IRS 2 is:

26 $$E^{r} = \sum\limits_{{m_{2} = 1 - \frac{{M_{2} }}{2}}}^{{\frac{{M_{2} }}{2}}} {\sum\limits_{{n_{2} = 1 - \frac{{N_{2} }}{2}}}^{{\frac{{N_{2} }}{2}}} {E_{{n_{2} ,m_{2} }}^{r} } }$$

The total signal power received by the user through the double reflection link is:

27 $$\begin{aligned} P_{r} & = \frac{{\left| {E^{r} } \right|^{2} }}{{2Z_{0} }}A_{r} \\ & = \frac{{P_{t} G_{t} G_{r} G_{1} G_{2} d_{x}^{2} d_{y}^{2} \lambda^{2} }}{{256\pi^{4} }} \\ & \times \left| {\sum\limits_{{m_{1} = 1 - \frac{{M_{1} }}{2}}}^{{\frac{{M_{1} }}{2}}} {\sum\limits_{{n_{1} = 1 - \frac{{N_{1} }}{2}}}^{{\frac{{N_{1} }}{2}}} {\frac{{\sqrt {F^{tx1} \left( {\theta_{{n_{1} ,m_{1} }}^{tx1} ,\varphi_{{n_{1} ,m_{1} }}^{tx1} } \right)F\left( {\theta_{{n_{1} ,m_{1} }}^{t1} ,\varphi_{{n_{1} ,m_{1} }}^{t1} } \right)F^{tx2} \left( {\theta_{{n_{12} ,m_{12} }}^{tx12} ,\varphi_{{n_{12} ,m_{12} }}^{tx12} } \right)F\left( {\theta_{{n_{12} ,m_{12} }}^{t12} ,\varphi_{{n_{12} ,m_{12} }}^{t12} } \right)} }}{{r_{{n_{1} ,m_{1} }}^{t1} r_{{n_{1} ,m_{1} }}^{t2} }}} } } \right|^{2} \\ & \times \left| {\sum\limits_{{m_{2} = 1 - \frac{{M_{2} }}{2}}}^{{\frac{{M_{2} }}{2}}} {\sum\limits_{{n_{2} = 1 - \frac{{N_{2} }}{2}}}^{{\frac{{N_{2} }}{2}}} {\frac{{\sqrt {F^{rx} \left( {\theta_{{n_{2} ,m_{2} }}^{rx} ,\varphi_{{n_{2} ,m_{2} }}^{rx} } \right)F\left( {\theta_{{n_{2} ,m_{2} }}^{r} ,\varphi_{{n_{2} ,m_{2} }}^{r} } \right)} }}{{r_{{n_{2} ,m_{2} }}^{r} }}e^{{ - j\left( {\frac{2\pi }{\lambda }r_{{n_{2} ,m_{2} }}^{r} - \phi_{{n_{2} ,m_{2} }} } \right)}} } } } \right|^{2} \\ \end{aligned}$$

Assuming that the peak radiation direction of the signal reflected by *U*(*n*_1_,*m*_1_) points to the center of IRS 2, *F*^*tx2*^($\theta_{{n_{{{1}2}} ,m_{{{1}2}} }}^{{tx{1}2}}$,$\varphi_{{n_{{{1}2}} ,m_{{{1}2}} }}^{{tx{1}2}}$)≈1 in far-field case. If $\phi_{{n_{2} ,m_{2} }}$=2π $r_{{n_{2} ,m_{2} }}^{r}$/*λ*, the received signal power of the user is maximized, as shown in Eq. (28).

28 $$\begin{aligned} P_{r}^{\max } & = \frac{{P_{t} G_{t} G_{r} G_{1} G_{2} d_{x}^{2} d_{y}^{2} \lambda^{2} }}{{256{\uppi }^{4} }} \\ & \times \left| {\sum\limits_{{m_{1} = 1 - \frac{{M_{1} }}{2}}}^{{\frac{{M_{1} }}{2}}} {\sum\limits_{{n_{1} = 1 - \frac{{N_{1} }}{2}}}^{{\frac{{N_{1} }}{2}}} {\frac{{\sqrt {F^{tx1} \left( {\theta_{{n_{1} ,m_{1} }}^{tx1} ,\varphi_{{n_{1} ,m_{1} }}^{tx1} } \right)F\left( {\theta_{{n_{1} ,m_{1} }}^{t1} ,\varphi_{{n_{1} ,m_{1} }}^{t1} } \right)F\left( {\theta_{{n_{12} ,m_{12} }}^{t12} ,\varphi_{{n_{12} ,m_{12} }}^{t12} } \right)} }}{{r_{{n_{1} ,m_{1} }}^{t1} r_{{n_{1} ,m_{1} }}^{t2} }}} } } \right|^{2} \\ & \times \left| {\sum\limits_{{m_{2} = 1 - \frac{{M_{2} }}{2}}}^{{\frac{{M_{2} }}{2}}} {\sum\limits_{{n_{2} = 1 - \frac{{N_{2} }}{2}}}^{{\frac{{N_{2} }}{2}}} {\frac{{\sqrt {F^{rx} \left( {\theta_{{n_{2} ,m_{2} }}^{rx} ,\varphi_{{n_{2} ,m_{2} }}^{rx} } \right)F\left( {\theta_{{n_{2} ,m_{2} }}^{r} ,\varphi_{{n_{2} ,m_{2} }}^{r} } \right)} }}{{r_{{n_{2} ,m_{2} }}^{r} }}} } } \right|^{2} \\ \end{aligned}$$

## Results and discussion

### Simulation scenarios

**Figure 3:**
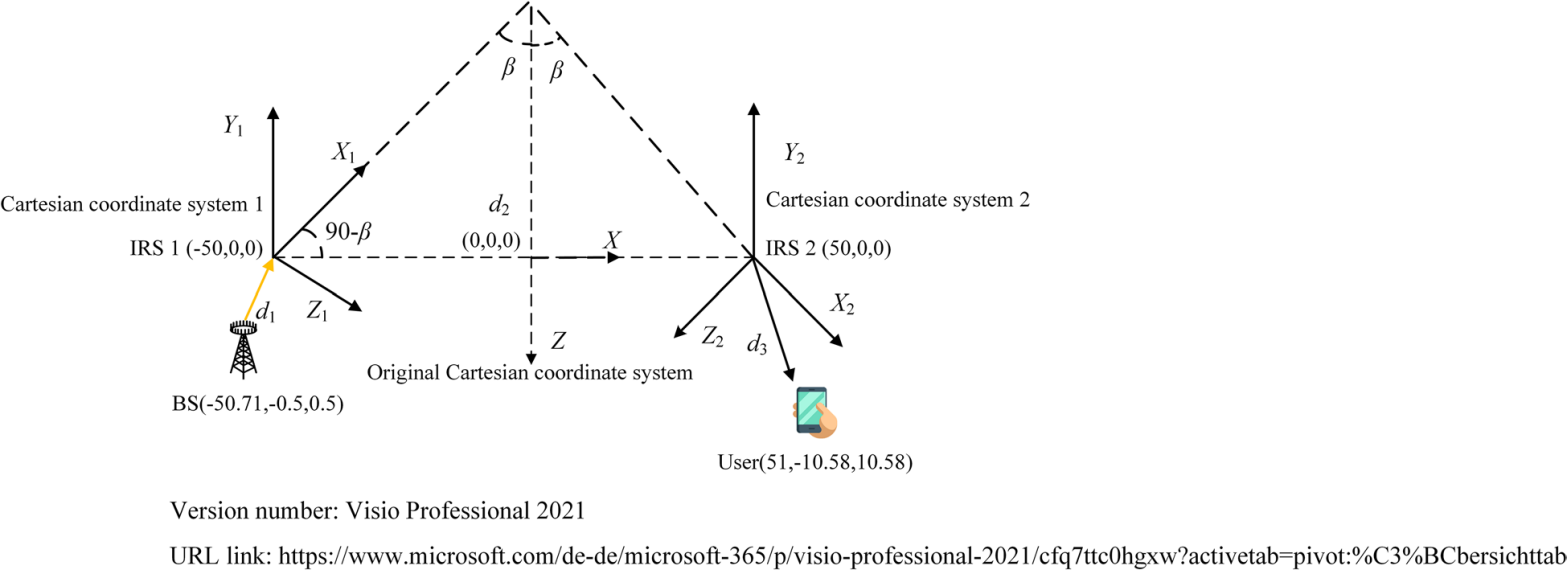
Simulation scenarios.

As shown in Fig. 3, the original Cartesian coordinate system is established whose origin is aligned with the midpoint of the connecting line between the centers of IRS 1 and IRS 2, and the positive *X* axis is horizontal right along the connecting line. In order to apply the path loss model proposed in Eq. (28) conveniently, the original coordinates of the BS and the user are pre-multiplied by rotation matrices and converted into the coordinates in Cartesian coordinate systems 1 and 2, respectively, as shown in Eqs. (29) to (32).

29 $$\left[ {\begin{array}{*{20}c} {x^{\prime}_{BS} } \\ {y^{\prime}_{BS} } \\ {z^{\prime}_{BS} } \\ 1 \\ \end{array} } \right] = \left[ {\begin{array}{*{20}c} {\cos \left( {\frac{{{\uppi }\left( {270 + \beta } \right)}}{180}} \right)} & 0 & {\sin \left( {\frac{{{\uppi }\left( {270 + \beta } \right)}}{180}} \right)} & 0 \\ 0 & 1 & 0 & 0 \\ { - \sin \left( {\frac{{{\uppi }\left( {270 + \beta } \right)}}{180}} \right)} & 0 & {\cos \left( {\frac{{{\uppi }\left( {270 + \beta } \right)}}{180}} \right)} & 0 \\ 0 & 0 & 0 & 1 \\ \end{array} } \right] \times \left[ {\begin{array}{*{20}c} {x_{BS} } \\ {y_{BS} } \\ {z_{BS} } \\ 1 \\ \end{array} } \right]$$

30 $$\left[ {\begin{array}{*{20}c} {x^{\prime}_{User} } \\ {y^{\prime}_{User} } \\ {z^{\prime}_{User} } \\ 1 \\ \end{array} } \right] = \left[ {\begin{array}{*{20}c} {\cos \left( {\frac{{{\uppi }\left( {270 + \beta } \right)}}{180}} \right)} & 0 & {\sin \left( {\frac{{{\uppi }\left( {270 + \beta } \right)}}{180}} \right)} & 0 \\ 0 & 1 & 0 & 0 \\ { - \sin \left( {\frac{{{\uppi }\left( {270 + \beta } \right)}}{180}} \right)} & 0 & {\cos \left( {\frac{{{\uppi }\left( {270 + \beta } \right)}}{180}} \right)} & 0 \\ 0 & 0 & 0 & 1 \\ \end{array} } \right] \times \left[ {\begin{array}{*{20}c} {x_{User} } \\ {y_{User} } \\ {z_{User} } \\ 1 \\ \end{array} } \right]$$

where ($x^{\prime}_{BS}$,$y^{\prime}_{BS}$,$z^{\prime}_{BS}$) and ($x^{\prime}_{User}$,$y^{\prime}_{User}$,$z^{\prime}_{User}$) are the coordinates of the BS and the user in Cartesian coordinate system 1, respectively. *β* is the deviation angle from the positive *X* axis of Cartesian coordinate system 1 to the negative *Z* axis of the original Cartesian coordinate system.

31 $$\left[ {\begin{array}{*{20}c} {x^{\prime\prime}_{BS} } \\ {y^{\prime\prime}_{BS} } \\ {z^{\prime\prime}_{BS} } \\ 1 \\ \end{array} } \right] = \left[ {\begin{array}{*{20}c} {\cos \left( {\frac{{{\uppi }\left( {90 - \beta } \right)}}{180}} \right)} & 0 & {\sin \left( {\frac{{{\uppi }\left( {90 - \beta } \right)}}{180}} \right)} & 0 \\ 0 & 1 & 0 & 0 \\ { - \sin \left( {\frac{{{\uppi }\left( {90 - \beta } \right)}}{180}} \right)} & 0 & {\cos \left( {\frac{{{\uppi }\left( {90 - \beta } \right)}}{180}} \right)} & 0 \\ 0 & 0 & 0 & 1 \\ \end{array} } \right] \times \left[ {\begin{array}{*{20}c} {x_{BS} } \\ {y_{BS} } \\ {z_{BS} } \\ 1 \\ \end{array} } \right]$$

32 $$\left[ {\begin{array}{*{20}c} {x^{\prime\prime}_{User} } \\ {y^{\prime\prime}_{User} } \\ {z^{\prime\prime}_{User} } \\ 1 \\ \end{array} } \right] = \left[ {\begin{array}{*{20}c} {\cos \left( {\frac{{{\uppi }\left( {90 - \beta } \right)}}{180}} \right)} & 0 & {\sin \left( {\frac{{{\uppi }\left( {90 - \beta } \right)}}{180}} \right)} & 0 \\ 0 & 1 & 0 & 0 \\ { - \sin \left( {\frac{{{\uppi }\left( {90 - \beta } \right)}}{180}} \right)} & 0 & {\cos \left( {\frac{{{\uppi }\left( {90 - \beta } \right)}}{180}} \right)} & 0 \\ 0 & 0 & 0 & 1 \\ \end{array} } \right] \times \left[ {\begin{array}{*{20}c} {x_{User} } \\ {y_{User} } \\ {z_{User} } \\ 1 \\ \end{array} } \right]$$

where ($x^{\prime\prime}_{BS}$,$y^{\prime\prime}_{BS}$,$z^{\prime\prime}_{BS}$) and ($x^{\prime\prime}_{User}$,$y^{\prime\prime}_{User}$,$z^{\prime\prime}_{User}$) are the corresponding coordinates of the BS and the user in Cartesian coordinate system 2, respectively.

To achieve a fair comparison between dyadic backscatter channel model- and spatial scattering channel model-based double IRSs-aided wireless communication systems, the simulation setup in^21^ is utilized, and the detailed settings are listed in Table 2. According to^19^, when the size of an IRS element along the *X* axis and *Y* axis *d*_*x*_  = *d*_*y*_  = 0.03 m and the carrier wavelength *λ*  = 0.06 m, the gain of the IRS element is about 4. Therefore, its normalized power radiation pattern is defined as *F*(*θ*,*φ*)  = cos*θ*. Both the BS transmit antenna and the receiving antenna of the user are assumed to be omnidirectional, and their normalized power radiation patterns are defined as *F*^*tx*^(*θ*,*φ*)  = *F*^*rx*^(*θ*,*φ*)  = 1. In addition, whether deploying double IRSs will enhance the system performance is explored by comparing with single IRS-aided wireless communication systems. For single IRS-aided wireless communication systems, the user is served by the BS through the single reflection link via IRS 2. In this case, the BS is in the far field of IRS 2, and according to^9^, the total received signal power at the user is:

33 $$P_{r2} = \frac{{P_{t} G_{t} G_{r} G_{2} d_{x} d_{y} \lambda^{2} F^{tx3} \left( {\theta_{tx3} ,\varphi_{tx3} } \right)F\left( {\theta_{t3} ,\varphi_{t3} } \right)}}{{64{\uppi }^{3} }} \times \left| {\sum\limits_{{m_{2} = 1 - \frac{{M_{2} }}{2}}}^{{\frac{{M_{2} }}{2}}} {\sum\limits_{{n_{2} = 1 - \frac{{N_{2} }}{2}}}^{{\frac{{N_{2} }}{2}}} {\frac{{\sqrt {F^{rx} \left( {\theta_{{n_{2} ,m_{2} }}^{rx} ,\varphi_{{n_{2} ,m_{2} }}^{rx} } \right)F\left( {\theta_{{n_{2} ,m_{2} }}^{r} ,\varphi_{{n_{2} ,m_{2} }}^{r} } \right)} }}{{r_{{n_{2} ,m_{2} }}^{t3} r_{{n_{2} ,m_{2} }}^{r} }}} } } \right|^{2}$$

where *θ*_*tx*3_ and *φ*_*tx*3_ are the elevation and azimuth angles from the BS transmit antenna to the center of IRS 2, respectively. Similarly, *θ*_*t*3_ and *φ*_*t*3_ are the reversed elevation and azimuth angles, respectively. $r_{{n_{2} ,m_{2} }}^{t3}$ is the Euclidean distance between the BS and *U*(*n*_2_,*m*_2_), and $r_{{n_{2} ,m_{2} }}^{t3}$≈*d*_4_ − sin*θ*_*t*3_cos*φ*_*t*3_(*m*_2_ − 1/2)*d*_*x*_ − sin*θ*_*t*3_sin*φ*_*t*3_(*n*_2_ − 1/2)*d*_*y*_. Here, *d*_4_ is the Euclidean distance from the BS to the center of IRS 2. $r_{{n_{2} ,m_{2} }}^{r}$ is the Euclidean distance between *U*(*n*_2_,*m*_2_) and the user. For a fair comparison, IRS 2 is configured with 800/1600 elements in above single IRS-aided wireless communication systems while IRS 1 and IRS 2 are configured with 800/1600 elements in total in double IRSs-aided wireless communication systems.

**Table 2:** Simulation parameter settings.

Parameters	Values
Euclidean distance between the BS and the center of IRS 1 *d*_1_	1 m/15 m
Euclidean distance between the centers of IRS 1 and IRS 2 *d*_2_	100 m
Euclidean distance between the center of IRS 2 and the user *d*_3_	15 m
Wavelength of the transmit signal *λ*	0.06 m
Size of each IRS element *d*_*x*_ × *d*_*y*_	0.03 m × 0.03 m
Transmit power of the BS* P*_*t*_	43 dBm
Noise power *σ*^2^	− 60 dBm
Deviation angle of IRS 1/IRS 2 *β*	45°
Gain of each IRS element *G*_1_/*G*_2_	4
Total number of IRS elements *K*	800/1600

### Simulation results and analysis

**Figure 4:**
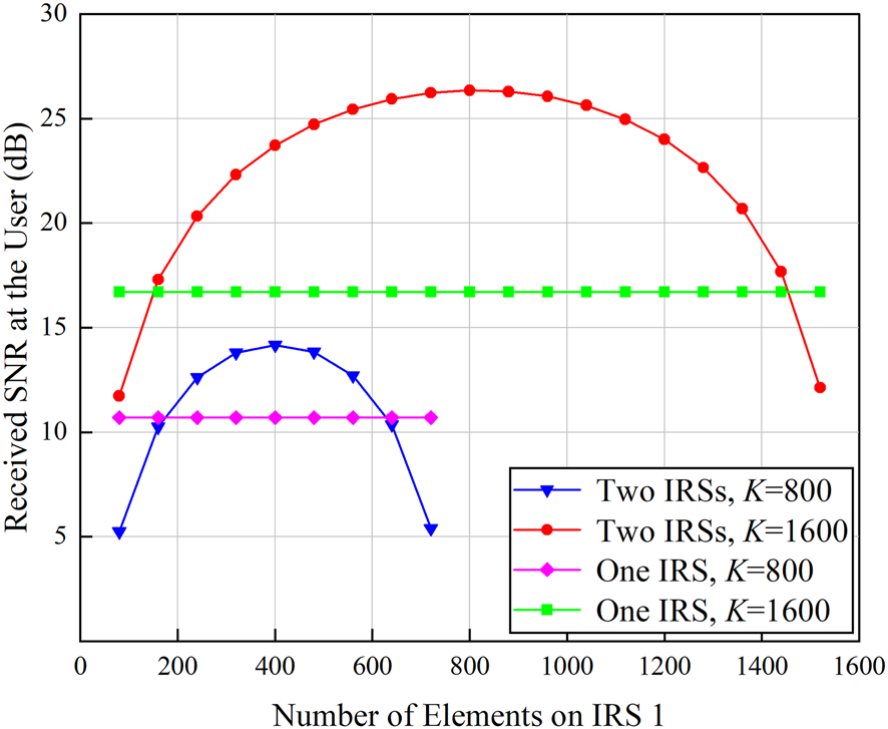
Received SNR at the user versus the number of elements on IRS 1 in the near-field case (*d*_1_  = 1 m).

The number of elements on IRS 1 is gradually increased while the total number of IRS elements *K* is kept unchanged to observe the received SNR at the user, and the results are shown in Fig. 4.

As can be observed from Fig. 4, the received SNR at the user is maximized when the same number of elements is assigned to IRS 1 and IRS 2. When *K* increases from 800 to 1600, the gain of single IRS-aided wireless communication systems is about 6 dB, i.e., the received power is improved by 4 times. Double IRSs-aided wireless communication systems can achieve about 12 dB gain, which means that the received signal power is enhanced by 16 folds. Therefore, compared with the array gain of $\mathcal{O}$(*K*^2^) brought by single IRS, double cooperative IRSs can provide a received power gain of $\mathcal{O}$(*K*^4^). The above conclusions are consistent with^21^. However, different from the conclusions drawn from^21^, that is, when *K* = 800, double IRSs-aided wireless communication systems are inferior to their single IRS-aided competitors, the simulation results in this paper show that even though *K*  = 800, double IRSs-aided wireless communication systems still gain advantages over single IRS-aided wireless communication systems. To be specific, when *d*_1_ = 1 m, the BS is in the near field of IRS 1, and as the number of elements on IRS 1 increases, more signal power can be received by IRS 1 from the BS. Correspondingly, the received SNR at the user gradually increases, and double cooperative IRSs can bring in about 3 dB power gain. As the number of elements on IRS 1 further increases, although IRS 1 can reflect more power towards IRS 2, the decrease of the number of elements on IRS 2 results in the decline of the received signal power. Therefore, the received SNR at the user is reduced. In addition, when *K* = 1600, deploying double cooperative IRSs can achieve a 9 dB gain which is higher than the 6 dB gain presented in^21^. The reasons can be explained as below: the conclusion drawn from^21^ is based on dyadic backscatter channel model which omits various factors such as the size and gain of IRS elements and the near/far-field effects of IRS, and as a result, the actual performance gain brought by double IRSs cannot be quantified accurately. In this paper, spatial scattering channel model is applied to model the path loss of the double reflection link, and more accurate performance analysis can be achieved.

**Figure 5:**
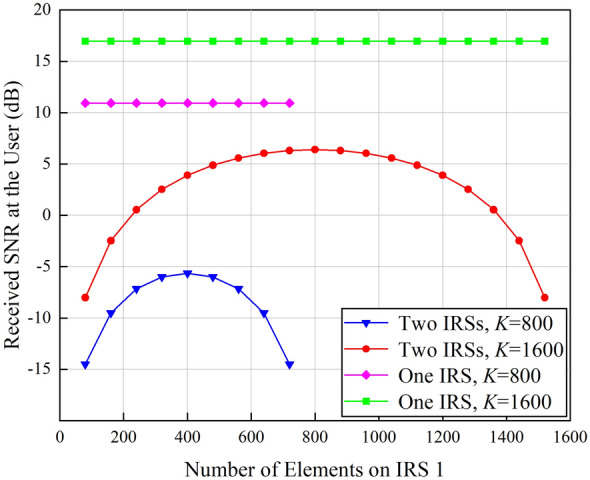
Received SNR at the user versus the number of elements on IRS 1 in the far-field case (*d*_1_  = 15 m).

In order to further explore the impact of near/far field effects of IRS on the received SNR, the distance between the BS and IRS 1 is enlarged, and it is equal to or larger than the distance between the user and IRS 2. In this case, the BS is in the far field of IRS 1. The above simulations are repeated, and the obtained results are shown in Fig. 5.

As shown in Fig. 5, similar to the results obtained in the near-field case, the received SNR at the user is maximized when the same number of elements is assigned to IRS 1 and IRS 2. However, even though the number of elements on IRS 1 keeps increasing, their array gain still cannot compensate for the path loss of far-field signal propagation. According to Eq. (28), if other parameters are kept unchanged, the path loss between the BS and IRS 1 in far-field case is 225 times higher than that in near-field case, and severe path loss leads to lower received SNR.

**Figure 6:**
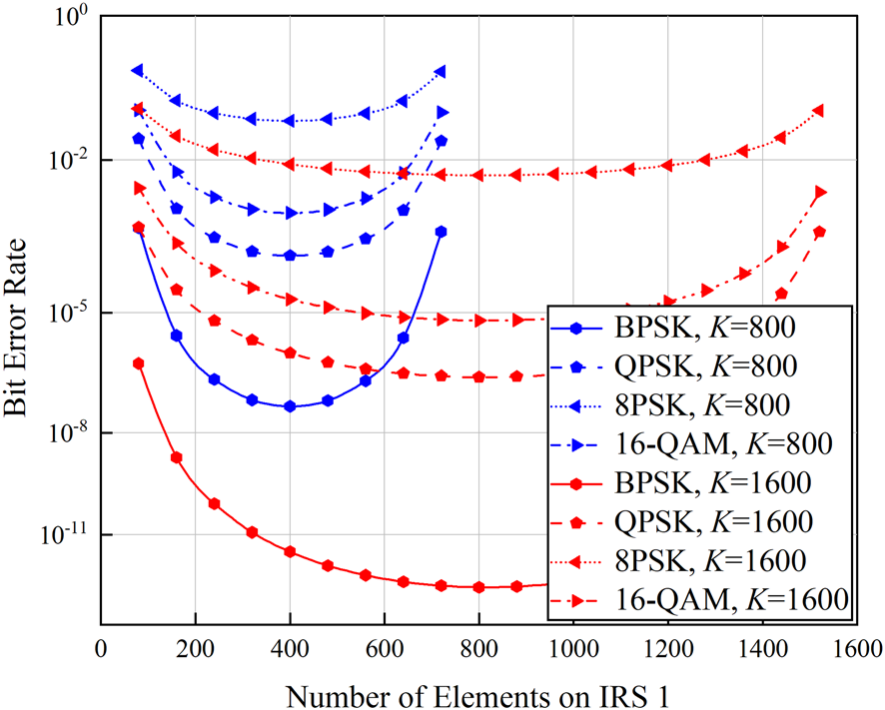
BER versus the number of elements on IRS 1.

Apart from the received SNR at the receiver, BER is also an important performance indicator of digital communication systems, and it highly depends on the input SNR of the demodulator with Gaussian white noise. Assuming that the channels in the cooperative double IRSs-aided wireless communication system are constant-parameter channels with ideal rectangular transmission characteristics within the frequency range of the signal. The channel noise is additive Gaussian white noise, and it affects the received signal only at the receiver side. Motivated by^27–33^, the BER performance of cooperative double IRSs-aided wireless communication system under BPSK, QPSK, 8PSK and 16-QAM modulation and coherent demodulation is investigated, and the simulation results are shown in Fig. 6.

As shown in Fig. 6, we can observe that the cooperative double IRSs-aided wireless communication system with BPSK modulation achieves its optimal BER performance when the total number of IRS elements is equally assigned to IRS 1 and IRS 2. This conclusion holds for other modulation methods, and the reason is analyzed as below: the received SNR at the user increases as the number of elements on IRS 1 increases, which results in a declined BER. The BER will increase as the phase difference between adjacent carriers decreases, which explains why the BER achieved by low-order phase-shift keying modulation is lower than high-order phase-shift keying modulation. In addition, the Euclidean distance between the 16-QAM constellation points falls in between that of the QPSK and 8PSK. Therefore, the BER performance of cooperative double IRSs-aided wireless communication system based on 16-QAM modulation method is higher than QPSK but lower than 8PSK.

**Figure 7:**
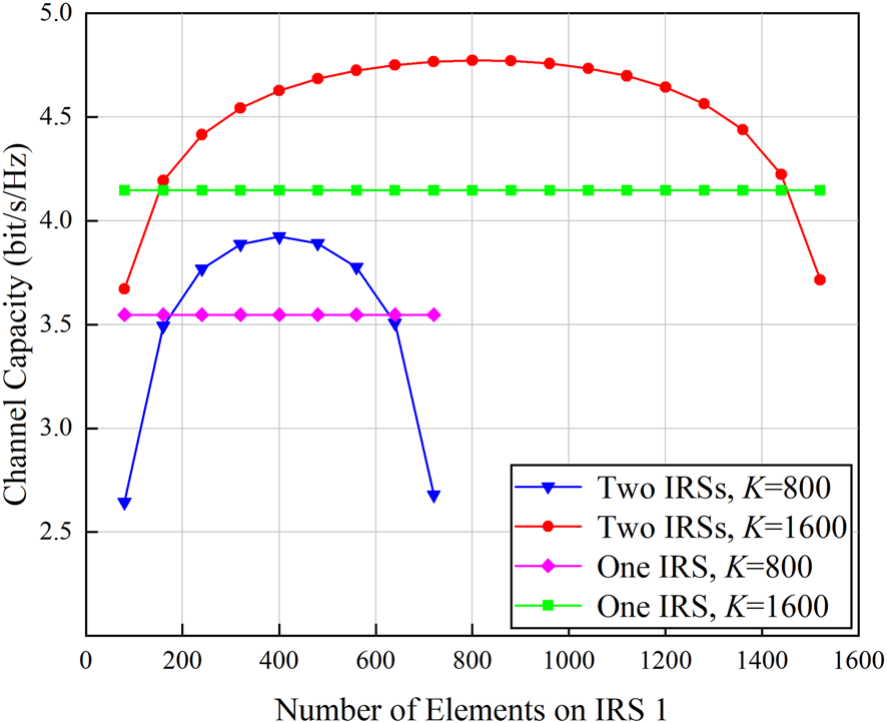
Channel capacity versus the number of elements on IRS 1.

Assuming that the channels in cooperative double IRSs-aided wireless communication system are bandwidth-constraint and they are affected by additive and continuous Gaussian white noise. According to the Shannon's law, the channel capacity of the cooperative double IRSs-aided wireless communication system is calculated when different number of IRS elements is assigned to IRS 1, and the results are shown in Fig. 7.

As shown in Fig. 7, the channel capacity of the cooperative double IRSs-aided wireless communication system first increases and then decreases as the number of elements on IRS 1 increases, and it achieves the maximum value when the number of elements on IRS 1 and IRS 2 are equal to each other. Its variation tendency can be analyzed from the change of the received SNR at the user, as shown in Fig. 4. When the total number of IRS elements increases from 800 to 1600, the channel capacity of the single IRS-aided wireless communication system is improved by 0.6 bit/s/Hz, and the channel capacity of the cooperative double IRSs-aided wireless communication system is increased by 0.9 bit/s/Hz under the optimal configuration. When the total number of IRS elements *K*  =  1600, the channel capacity improvement gained by the cooperative double IRSs-aided wireless communication system over its single IRS counterpart is twice the performance enhancement under *K* = 800. The above simulation results are consistent with the above drawn conclusions, that is, the performance gain achieved by cooperative double IRSs-aided wireless communication system depends on practical network configurations.

**Figure 8:**
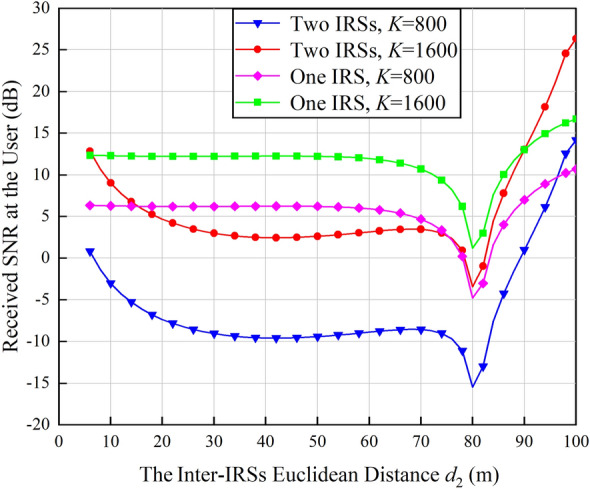
Received SNR at the user versus the inter-IRSs Euclidean distance *d*_2_.

As shown in Eq. (28), when the transmit power, antenna gains, carrier wavelength and the size of IRS are fixed, the total received signal power is only related to the Euclidean distances *d*_1_, *d*_2_ and *d*_3_ which are determined by the positions of double IRSs. In order to identify the optimal positions of the double IRSs, we observe the variations of the received SNR at the user versus the inter-IRSs Euclidean distance *d*_2_. The positions of the BS and the user are fixed while IRS 1 and IRS 2 move along the *X* axis in the original Cartesian coordinate system. For convenience, they are symmetric about the origin, i.e., the coordinate of IRS 1 in the original Cartesian coordinate system is (− *x*, 0, 0) and that of IRS 2 is (*x*, 0, 0). In this case, *d*_2_ = 2*x*. According to^21^, when IRS 1 is in the far field of IRS 2, if IRS 1 beams towards one element on IRS 2, the rest elements on IRS 2 can enjoy the same power gain. However, when IRS 1 is in the near field of IRS 2, the IRS coefficient adjustment adopted cannot align all the signals, which results in performance degradation. Therefore, in order to guarantee that IRS 1 is in the far field of IRS 2, *d*_2_ is at least 6 m. IRS 1 and IRS 2 are configured with the optimal number of reflecting elements, i.e., all elements are divided equally between IRS 1 and IRS 2. Changing *x* within its feasible set *x* ∈ (3, 50], the received SNR at the user is shown in Fig. 8.

As shown in Fig. 8, since the path loss of double IRSs-aided wireless communication system is approximately *d*_2_ squared times higher than that of single IRS-aided system, the received SNR at the user in double IRSs-aided wireless communication system changes faster as *d*_2_ increases. Specifically, when the total number of IRS elements is *K*=1600, the performance gain achieved by the double IRSs-aided wireless communication system is higher than its single IRS counterpart when *d*_2_ is larger than 90 m; When *K*=800, *d*_2_ needs to be larger than 96 m to guarantee the higher performance of double IRSs-aided wireless communication system. They both achieve the highest performance gain when *d*_2_=100 m. This means that the optimal positions of the double IRSs are (− 50, 0, 0) and (50, 0, 0), which are exactly the original simulation setups in Table 2.

In all, compared with single IRS-aided wireless communication systems, the performance gain brought by double IRSs-aided wireless communication systems is closely related to factors such as the number of IRS elements and the location of IRSs. Whether two cooperative IRSs should be adopted needs to be determined based on practical network configurations. In addition, if double IRSs are applied and other links are seriously blocked by obstacles, they should be assigned with the same number of elements to maximize the system performance.

## Conclusions

Focusing on the typical application scenarios of double IRSs-aided wireless communication systems, spatial scattering channel model is firstly leveraged to quantify the path loss of the double reflection link and establish the quantitative relationship between the received signal power and various system parameters. The impact of near/far-field effects of IRS on signal propagation is further taken into consideration to recognize the network configurations under which double cooperative IRSs can enhance the system performance. Simulation results show that the system performance is maximized when the two IRSs are assigned with the same number of elements. Compared with the array gain of $\mathcal{O}$(*K*^2^) brought by single IRS, cooperative double IRSs can achieve a power gain of $\mathcal{O}$(*K*^4^). Specifically, in the near-field case, even with a small number of total elements, i.e., 800, the performance gain achieved by the proposed double IRSs-aided wireless communication system based on the spatial scattering channel modeling is 3 dB higher than that of dyadic backscattering model-based system under the same parameter configurations. In addition, the channel capacity gain of the double IRSs-aided wireless communication system is twice the gain of single IRS-aided system when the total number of elements is increased from 800 to 1600. Moreover, the path loss is jointly determined by IRS properties and transmission distances. In the far-field case, since the path loss is heavily sustained by the double reflection link, and it is always *d*_2_ squared times higher than that of the single IRS-aided system. As a result, double IRSs-aided wireless communication systems are inferior to their single IRS-aided competitors. The above conclusions are drawn with the assumption that other links are seriously blocked by obstacles. Next, we will focus on the scenarios where all links between the transmitter and the receiver are unblocked to explore the full potentials of double IRSs-aided wireless communication systems.

## Data Availability

The datasets generated during and/or analysed during the current study are available from the corresponding author on reasonable request.
